# Gadolinium-Doped Hydroxyapatite Nanoparticles Functionalized with Curcumin and Folic Acid: Structural Insights and Magnetic Behavior for Theranostic Applications

**DOI:** 10.3390/ma19030449

**Published:** 2026-01-23

**Authors:** Jéssica P. N. Marinho, Luísa A. F. Vieira, André F. Oliveira, Aloísio M. Garcia, Monica E. B. Guarin, João Batista S. Barbosa, Yan F. X. Ladeira, Adolfo H. M. Silva, Edésia M. B. de Sousa

**Affiliations:** 1 Centro de Desenvolvimento da Tecnologia Nuclear (CDTN), Belo Horizonte 31270-901, MG, Brazil; jessica.marinho@cdtn.br (J.P.N.M.); luisa.vieira@cdtn.br (L.A.F.V.); afo@cdtn.br (A.F.O.); aloisio.garcia@cdtn.br (A.M.G.); monica.guarin@cdtn.br (M.E.B.G.); jbsb@cdtn.br (J.B.S.B.); 2 Departamento de Química, Universidade Federal de Minas Gerais (UFMG), Belo Horizonte 31270-901, MG, Brazil; yan.ximenes.012@gmail.com (Y.F.X.L.); adolfohmoraes@ufmg.br (A.H.M.S.)

**Keywords:** hydroxyapatite nanoparticles, gadolinium doping, curcumin functionalization, folic acid targeting, microwave-assisted synthesis, theranostic nanomaterials

## Abstract

Gadolinium-doped hydroxyapatite nanoparticles (HapGd NPs) have emerged as promising multifunctional platforms for biomedical applications due to their unique combination of biocompatibility, structural tunability, and magnetic responsiveness. In this work, HapGd nanoparticles were synthesized using a microwave-assisted method and subsequently functionalized with curcumin and folic acid to enhance therapeutic efficiency and selective targeting. The synthesized nanostructures were characterized using various techniques, including X-ray diffraction (XRD), transmission electron microscopy (TEM), Fourier-transform infrared spectroscopy (FTIR), thermogravimetric analysis (TGA), vibrating sample magnetometry (VSM), and relaxometry. Structural analyses revealed successful incorporation of Gd^3+^ ions into the Hap lattice, resulting in reduced unit cell volume and slight lattice distortion, while preserving the apatite crystalline framework. Surface functionalization with curcumin and folic acid was confirmed through spectroscopic characterization, demonstrating effective molecular attachment. Nuclear Magnetic Resonance (NMR) relaxation measurements indicated that Gd doping endowed paramagnetic behavior suitable for contrast enhancement in magnetic resonance imaging (MRI). Relaxometry studies revealed a strong linear correlation between 1/T_1_ and the Gd^3+^ concentration, especially in the functionalized samples, with performance comparable to the commercial contrast agent Omniscan™. The developed HapGd-based nanoplatform exhibits integrated diagnostic and therapeutic potential, providing a foundation for future research in biomedical applications.

## 1. Introduction

Osteosarcoma is the most common primary malignant bone tumor, characterized by aggressive local growth, high metastatic potential, and limited therapeutic options, particularly in recurrent and chemoresistant cases [1,2]. In this context, the development of multifunctional nanomaterials that combine therapeutic and diagnostic capabilities has attracted increasing attention for bone cancer management. These nanosystems are designed to integrate targeted drug delivery and imaging functionalities, improving treatment precision and reducing systemic toxicity compared to conventional chemotherapeutic approaches [3,4]. Among the wide variety of materials explored for this purpose, hydroxyapatite (Hap) stands out due to its chemical and structural similarity to bone mineral, as well as its biocompatibility, osteoconductivity, and versatile surface chemistry that enables doping and functionalization [5].

One of the most promising strategies to enhance Hap functionality is the incorporation of lanthanide ions, particularly gadolinium (Gd^3+^). Gadolinium is a paramagnetic element that is widely used as a contrast agent in magnetic resonance imaging (MRI) [6]. However, conventional Gd-based chelates have raised safety concerns related to nephrogenic systemic fibrosis and long-term Gd accumulation in tissues [7]. In contrast, Gd-doped hydroxyapatite (HapGd) offers a safer and more stable alternative, as the Gd^3+^ ions are incorporated within the Hap crystal lattice, reducing the risk of release into biological environments while maintaining MRI contrast efficiency [8,9].

To complement the diagnostic potential of HapGd, therapeutic functions can be introduced through the addition of bioactive compounds, such as curcumin (CM), the main polyphenol derived from *Curcuma longa*. Curcumin exhibits strong antioxidant, anti-inflammatory, and anticancer activities; however, its clinical application is hindered by low water solubility, instability under physiological conditions, and rapid metabolism [10,11]. To address these issues, curcumin can be immobilized onto Hap surfaces using 3-aminopropyltriethoxysilane (APTES) coupling. This process improves its stability and binding affinity, enabling a gradual release of curcumin at the target site [12].

Recent studies have demonstrated that curcumin–hydroxyapatite composites exhibit bioactivity and therapeutic potential. Jogiya et al. (2018) [13] synthesized Cur–Hap nanocomposites by a surfactant-mediated approach. They observed a reduction in crystallite size, hydrogen-bond interactions between curcumin and phosphate groups, and enhanced bioactivity in simulated body fluid, along with significant antimicrobial effects and hemocompatibility [13]. Similarly, Sharifi et al. (2021) prepared hydroxyapatite–gelatin/curcumin nanofibrous composites via electrospinning, achieving uniform morphology, a two-stage curcumin release (60% within 2 days followed by sustained release up to 14 days), and pronounced antibacterial activity against *S. aureus*, *E. coli*, and *S. mutans* [14]. These findings highlight the versatility of curcumin-functionalized Hap systems for drug delivery and tissue regeneration, suggesting the need for further exploration in multifunctional nanoplatforms.

To further enhance the therapeutic efficiency of curcumin-based systems, folic acid (FA, vitamin B9) has been widely investigated as a targeting ligand due to its high affinity for folate receptors, which are overexpressed in various cancer cells, including those from osteosarcoma and cervical tumors [15,16]. The conjugation of curcumin with FA has been shown to improve its bioavailability and increase its selectivity toward malignant cells, thereby reducing systemic toxicity and enhancing antitumor efficacy [17]. For instance, Ramezani Farani et al. (2022) developed FA-adorned curcumin-loaded iron oxide nanoparticles for cervical cancer treatment, achieving high encapsulation efficiency (≈88%), selective internalization in HeLa cells, and significant tumor cell inhibition while maintaining biocompatibility and MRI contrast capability [18]. Similarly, Ghoreyshi et al. (2023) reported that folic acid-linked chitosan-coated PLGA-based curcumin nanoparticles enhanced cellular uptake, reduced oxidative stress markers, and increased the activity of antioxidant enzymes in glioblastoma cells [19]. These findings suggest that integrating FA into curcumin–Hap systems could further enhance targeted drug delivery and therapeutic performance, offering a promising strategy for developing multifunctional nanoplatforms for osteosarcoma treatment.

Several synthesis methods have been applied to produce doped hydroxyapatite, including sol–gel, precipitation, and hydrothermal techniques. Nevertheless, these processes often require long reaction times and high temperatures, which limit scalability and reproducibility. In contrast, microwave-assisted synthesis has emerged as a rapid, energy-efficient, and environmentally friendly route to obtain nanocrystalline Hap with uniform morphology, high purity, and tunable particle size. Şimşek and Avcı (2019) [20] successfully produced highly crystalline hydroxyapatite powders using microwave-assisted precipitation in simulated body fluid. Their findings demonstrated that microwave irradiation significantly reduced the reaction time to less than 10 min while enhancing crystallinity and achieving a Ca/P ratio close to that of natural bone (1.64). The authors attributed these effects to the efficient volumetric heating provided by microwaves, which accelerates ion diffusion and crystal nucleation, leading to homogeneous and reproducible nanostructures [20]. Similarly, Aziz et al. (2023) [21] synthesized silver-doped carbonate hydroxyapatite nanoparticles using a microwave-assisted precipitation method. They obtained nanocrystalline particles measuring between 17 and 28 nanometers, with a controlled composition, high antibacterial activity, and low cytotoxicity toward pre-osteoblast cells (>70% viability). Their results confirmed that microwave-assisted synthesis enables precise control over crystal size and dopant incorporation while preserving biocompatibility and functional properties [21].

Despite these advances, reports on the microwave-assisted synthesis of lanthanide-doped and curcumin-functionalized hydroxyapatite remain limited. This gap highlights the potential to combine this fast and uniform synthesis method with gadolinium doping and bioactive molecule functionalization to create multifunctional theranostic nanomaterials. In this study, we present the microwave-assisted synthesis and characterization of Gd-doped hydroxyapatite nanoparticles functionalized with curcumin, modified with APTES, and folic acid. The main hypothesis is that microwave processing promotes rapid and homogeneous nucleation, leading to nanostructures with enhanced crystallinity, stability, and magnetic response suitable for theranostic applications. The results indicate that this synthetic route offers a promising pathway to develop multifunctional, biocompatible nanosystems for future use in cancer diagnosis and therapy.

## 2. Materials and Methods

### 2.1. Materials

Calcium nitrate tetrahydrate, Ca(NO_3_)_2_.4H_2_O, (Sigma-Aldrich, St. Louis, MO, USA), potassium phosphate dibasic trihydrate, K_2_HPO_4_.3H_2_O, (Sigma-Aldrich), gadolinium nitrate hexahydrate, Gd(NO_3_)_3_.6H_2_O, (Sigma-Aldrich), ammonium hydroxide, NH_4_OH, (Sigma-Aldrich), curcumin—CM, [HOC_6_H_3_(OCH_3_)CH=CHCO]_2_CH_2_, (Sigma-Aldrich), 3-amino-propyltriethoxysilane—APTES, H_2_N(CH_2_)_3_Si(OC_2_H_5_)_3_, (Sigma-Aldrich), acetic acid P.A., CH_3_CO_2_H, (Neon, New York, NY, USA), folic acid, C_19_H_19_N_7_O_6_, (Sigma-Aldrich).

### 2.2. Synthesis of Hydroxyapatite Nanoparticles Using Microwaves

The synthesis of pure hydroxyapatite (Hap) nanoparticles and nanoparticles doped with different concentrations of gadolinium (Gd) by microwave irradiation was conducted based on the hydrothermal method, incorporating specific modifications to the conventional procedure. Initially, two precursor solutions were prepared: solution I, containing calcium (Ca^2+^) and gadolinium (Gd^3+^) ions, and solution II, containing phosphate (PO_4_^3−^) ions. The samples were obtained by partially replacing calcium with gadolinium at molar ratios of 0, 2.5, 5, 7.5, 10, and 15%, while maintaining the total concentration of precursor ions (Ca^2+^ + Gd^3+^) at 1 mol·L^−1^. Solution II, at a concentration of 0.6 mol·L^−1^, was prepared by dissolving diammonium hydrogen phosphate ((NH_4_)_2_HPO_4_) in deionized water, then adjusting the pH to 11 with ammonium hydroxide (NH_4_OH). Solution I was then added dropwise to Solution II at a rate of 0.5 mL·min^−1^ under constant stirring at room temperature. After completion of the addition, the resulting suspension was transferred to a microwave reactor.

The reaction was carried out without pressure control using a round-bottom flask coupled to a reflux system. Heating was maintained at 150 °C for 5 min, at a power of 300 W. After completion of the process, the material obtained was centrifuged, washed successively with deionized water and ethanol, and dried in an oven at 60 °C for 24 h. Subsequently, the resulting powder was calcined at 600 °C for 6 h.

The synthesized samples were designated as shown in Table 1.

### 2.3. Functionalization of Hap Nanoparticles with Curcumin

To incorporate curcumin into Hap and HapGd nanoparticles, curcumin (CM) was initially modified with APTES, as published in previous works [22]. In summary, the modification of curcumin with APTES was carried out in an acidic medium at 80 °C, in a reflux system with an argon atmosphere. After the reaction, the material was suspended in water and lyophilized, resulting in a powder material called CMAP.

The addition of CMAP into the nanoparticles was carried out directly by mixing CMAP with Hap and Gd-doped Hap samples, at a mass ratio of 1:1 in 50 mL of deionized water. The mixture was kept under stirring for 24 h at 50 °C. The resulting orange powder was collected by vacuum filtration, washed with deionized water and excess ethanol to remove the unincorporated CMAP. Subsequently, the material was dried in an oven at 60 °C for 24 h.

### 2.4. Functionalization of Nanostructures with Folic Acid

To obtain the nanocomposite for active targeting, folic acid was incorporated into the Hap nanostructure with CMAP. Analogous to the previous process, the synthesis was carried out by directly mixing Hap–CMAP and folic acid at a 1:1 mass ratio in water. The mixture was stirred at 50 °C for 24 h. After the reaction period, the material was washed with deionized water and ethanol, filtered, and dried in an oven at 60 °C for 24 h.

### 2.5. Characterization

#### 2.5.1. X-Ray Diffraction (XRD)

The crystalline structure of the synthesized materials was characterized by synchrotron powder X-ray diffraction (XRD), performed at 300 K at the Paineira beamline of the Brazilian Synchrotron Light Laboratory (LNLS-CNPEM, Campinas, Brazil). Measurements were performed at room temperature using a 5 mm diameter Kapton capillary. The selected X-ray wavelength was 0.551038 Å, corresponding to an energy of 22.5 keV. The Paineira diffractometer (LNLS-CNPEM, Campinas, Brazil) is a high-power, three-circle Newport diffractometer designed to operate in Debye–Scherrer geometry. In this instrument, detection was performed using a high-resolution Multi-Analyzer Crystals (MAC) (FMB-Oxford, Orford, OX2, UK) detector with an angular resolution of 0.008° and a 2Ɵ angular range of 3° to 145°. A complete diffraction pattern was obtained in the variable 2Ɵ range of 2° to 40°.

#### 2.5.2. Rietveld Refinement

The XRD data were analyzed using the GSAS-II software (V5.6.0), based on the Rietveld refinement method [23]. The crystal model used for refinements was based on the CIF code ICSD 26204 [24]. For the Gd substitution samples, the structural CIF was changed in the Ca position site occupation to maintain the chemical conditions. Rietveld refinement was carried out for Hap (0%) and Gd substitutions at 2.5%, 5%, 7.5%, and 10%. The peak profiles were fitted using a pseudo-Voigt profile function. The background was refined using a Chebyshev polynomial function for the pure Hap sample, and a combination of Chebyshev polynomials and manual fitting was used for the other cases. The zero shift, scale factor, sample displacement, cell parameters, preferential orientation, and microstrain were permitted to vary. The displacement parameters, atomic positions, and occupancies were kept constant throughout the refinement process. The average crystallite size estimate was obtained using JADE/MDI software version 9.7.

#### 2.5.3. Fourier Transform Infrared Spectroscopy (FTIR-ATR)

Characterization of the functional groups was performed by Fourier transform infrared spectroscopy (FTIR-ATR) on a Bruker Vertex 70v spectrometer (Bruker Scientific LLC., Billerica, MA, USA). Spectra were recorded in transmission mode using a platinum–diamond ATR accessory under vacuum, with 64 scans acquired at a spectral resolution of 4 cm^−1^, covering the range 4000–400 cm^−1^.

#### 2.5.4. Diffuse Reflectance Infrared Fourier Transform Spectroscopy (DRIFTS)

Diffuse reflectance infrared Fourier transform spectroscopy (DRIFTS) was performed using a Harrick accessory coupled to the Bruker FTIR-70V. Measurements were conducted in the spectral range of 4000 to 400 cm^−1^. Before measurement, the sample was manually ground in an agate mortar and subsequently sieved through a 38 μm mesh to reduce the diffusion coefficient. Also, for the measurement, the sample was mixed with KBr to reduce excessive sample concentration.

#### 2.5.5. Thermogravimetric Analysis (TGA)

Thermogravimetric analysis (TGA) was performed using a DTG-60H analyzer (Shimadzu, Kyoto, Japan) at 25–800 °C with a heating rate of 10 °C min^−1^. Measurements were performed under nitrogen at a flow rate of 100 mL min^−1^.

#### 2.5.6. Transmission Electron Microscopy (TEM)

Sample morphology was analyzed by transmission electron microscopy (TEM; Tecnai G2-12 Spirit Biotwin, FEI, 120 kV, FEI Company, Hillsboro, Oregon, USA), performed at the Microscopy Center of the Federal University of Minas Gerais (UFMG). To determine the average particle size, the ImageJ software (ImageJ, V 1.54k—U.S. National Institutes of Health, Bethesda, MD, USA) was used.

#### 2.5.7. Vibrating Sample Magnetometry (VSM)

The magnetic properties of the synthesized materials were investigated by vibrating sample magnetometry (VSM, 7400 series, LakeShore Cryotronics, Westerville, OH, USA).

#### 2.5.8. T_1_ Relaxometry and R_1_ Determination

Longitudinal relaxation times (T_1_) were measured to determine the relaxivity (R_1_) of the hydroxyapatite nanoparticles doped with 2.5% Gd^3+^ (HapGd2.5 and HapGd2.5-CMAP-FA) using the Bruker TD-NMR minispec mq20 (Bruker, BW, Ettlingen, Germany) of the Center of Education and Innovation in Chemistry (CEI) of the Department of Chemistry of UFMG. In this study, a single Gd loading was used because higher Gd contents hinder homogeneous dispersion, and the high-power sonication required to achieve it may detach loosely bound Gd^3+^, producing mixtures of free ions and Gd-doped nanoparticles that would compromise T_1_ accuracy. For each formulation, 126 mg of nanoparticles were suspended in 50 mL of deionized water and dispersed using an Ultronique Desruptor T5 sonicator (595 W, macrotip, Indaituba, SP, Brazil) under an optimized cycle of 3 s ON/3 s OFF for 20 min, a 15 min cooling interval, and another 20 min of sonication in an ice bath. This yielded reproducible stock suspensions containing 0.4 mM Gd^3+^. A Gd-free hydroxyapatite control was prepared identically. Stock solutions were diluted directly into 18 mm NMR tubes to obtain final Gd^3+^ concentrations of 0.025–0.20 mM in 1 mL. Then, 1 mL of a preheated 2% *w*/*v* agarose solution was added to form 1% agarose gels (final volume of 2 mL) [25]. Reference samples of Omniscan™ (GE Healthcare, São Paulo, SP, Brazil) (gadodiamide, 0.5 mmol·mL^−1^) were prepared similarly but without sonication. T_1_ measurements were performed on a Bruker Minispec mq20 (0.5 T, 20 MHz) at 40 °C using an inversion–recovery sequence (τ = 5–8000 ms; 25 points). Samples equilibrated in the probe for 10 min before acquisition. T_1_ values were obtained by monoexponentially fitting, and R_1_ was determined from linear plots of 1/T_1_ versus [Gd^3+^] [25].

## 3. Results and Discussion

### 3.1. X-Ray Diffraction (XRD)

**Figure 1:**
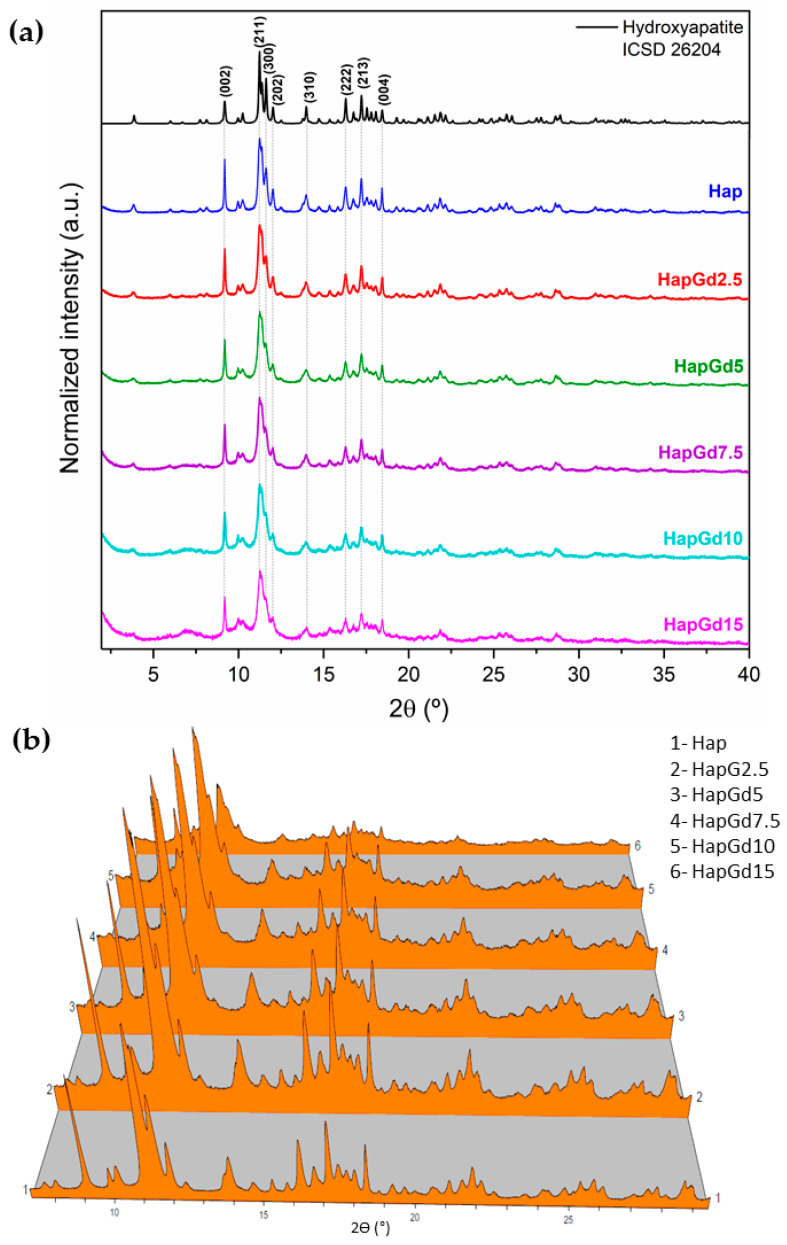
(**a**) XRPD patterns of samples, inset of 2Ɵ range 2° to 4°, (**b**) 3D representation of the diffractograms for better visualization of background increase and peak broadening.

Figure 1a shows diffractograms indicating that all samples exhibit the characteristic diffraction planes of hydroxyapatite (Hap) with hexagonal symmetry, consistent with the established crystallographic pattern of the P6_3_/m phase. The pure hydroxyapatite (Hap) sample exhibits well-defined peaks with high intensity and low background, indicating a high degree of crystallinity. As the gadolinium concentration increases, progressive peak broadening and baseline elevation become evident, indicating a progressive loss of structural order (Figure 1b).

**Figure 2:**
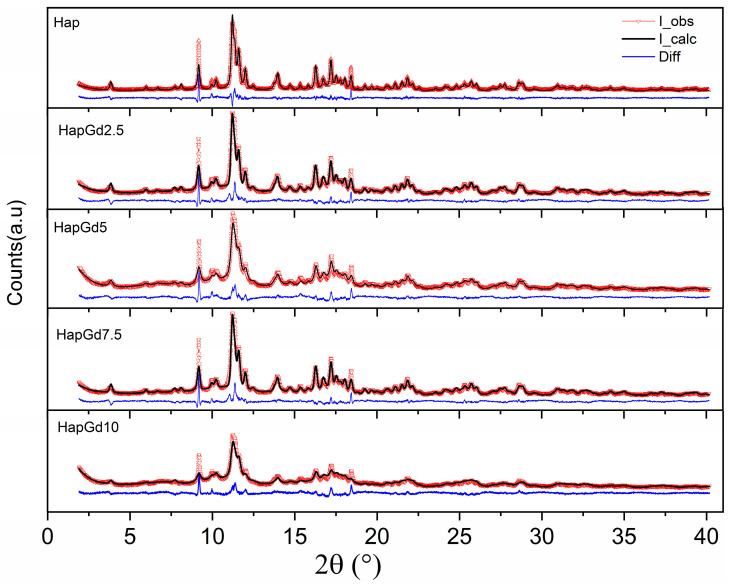
Experimental and calculated (dotted red line) patterns and observed patterns (solid black line), and difference (blue line).

The absence of additional reflections in all compositions demonstrates that no formation of secondary phases detectable by XRD occurs. This finding is reinforced by the Rietveld refinement results (Figure 2), which show that the crystallographic model based on the CIF ICSD 26204 file agrees well with the experimental data. The corresponding reliability factors (Table 2) fall within the commonly accepted ranges for comparable materials, thereby confirming the quality of the adjustments. The diffraction patterns in Figure 1 already indicate a reduction in crystalline quality, a trend further supported by the reliability factors. The sample containing 15% Gd was excluded from the Rietveld refinement due to the significant structural disorder and local heterogeneity induced by the dopant at this concentration. While the hydroxyapatite phase remains present, the coexistence of highly distorted regions at the nanoscale hinders an accurate representation of the material in the refinement process. Consequently, the refinement adjustments become unstable and display elevated reliability factors, thereby compromising both the quality and representativeness of the results. To deepen the understanding of the crystalline structure, especially at high Gd concentrations, complementary techniques such as Electron Paramagnetic Resonance (EPR) and Extended X-ray Absorption Fine Structure (EXAFS) can provide detailed information about the local environment and the crystallographic sites occupied by Gd^3+^ ions, and these analyses are planned for future steps in this work.

The refined unit cell parameters reveal a clear trend of decreasing crystal volume with increasing Gd doping. During the hydroxyapatite doping process, Ca^2+^ ions are partially substituted within the crystal structure. This cation occupies two distinct sites in apatite: Ca(I) and Ca(II). The Ca(I) site exhibits coordination by nine oxygen atoms of the phosphate groups, forming a tricapped trigonal prism polyhedron (CaO_9_) with C_3_ symmetry. The Ca(II) site exhibits distinct coordination, composed of six oxygens from the phosphate and a hydroxyl group, resulting in a CaO_6_OH polyhedron with C_s_ symmetry [26,27].

The introduction of trivalent rare earth ions, such as Gd^3+^, is structurally feasible due to the proximity between their ionic radii and those of Ca^2+^ at different coordination numbers. At coordination 9, the radius of Gd^3+^ (1.107 Å) is comparable to that of Ca^2+^ (1.18 Å), while at coordination 7 this relationship also holds (Ca^2+^ = 1.06 Å; Gd^3+^ ≈ 1.00 Å) [28]. Despite this dimensional similarity, the valence difference between Ca^2+^ and Ln^3+^ requires compensatory mechanisms to preserve the charge neutrality of the structure [29,30]. Two main compensation mechanisms are described for lanthanide-doped apatites. The first involves the formation of cationic vacancies, according to the equilibrium:

3Ca^2+^ ⇌ 2Ln^3+^ + V_Ca_, (1)

This process tends to occur when the dopant preferentially occupies the Ca(I) sites and may also reduce the (Ca + Ln)/P ratio of the structure. The second mechanism is associated with the substitution of hydroxyl groups by oxygen, favored at the Ca(II) site due to its proximity to the OH^−^ column of the apatite:

Ca^2+^ + OH^−^ ⇌ Ln^3+^ + O^2−^, (2)

Heterovalent substitution induces local lattice distortions related to charge compensation mechanisms, such as vacancy formation or anionic redistribution, which lead to a global contraction of the unit cell. The decrease in the c parameter is particularly indicative of disturbances in the structural column formed by Ca(II) ions.

Results showed that the unit cell volume decreased with the increase of gadolinium content. This increase in gadolinium (Gd) content was accompanied by broadening of the diffraction peaks and an increase in background intensity. A common interpretation would attribute this reduction to an enhanced contribution from an amorphous phase. However, reflections associated with the crystalline phase remained, with no indication of a secondary phase. While no significant increase in particle size was observed, the broadening of the peaks and elevated background suggest that the crystalline state may have been altered by a reduction in crystallite size.

**Figure 3:**
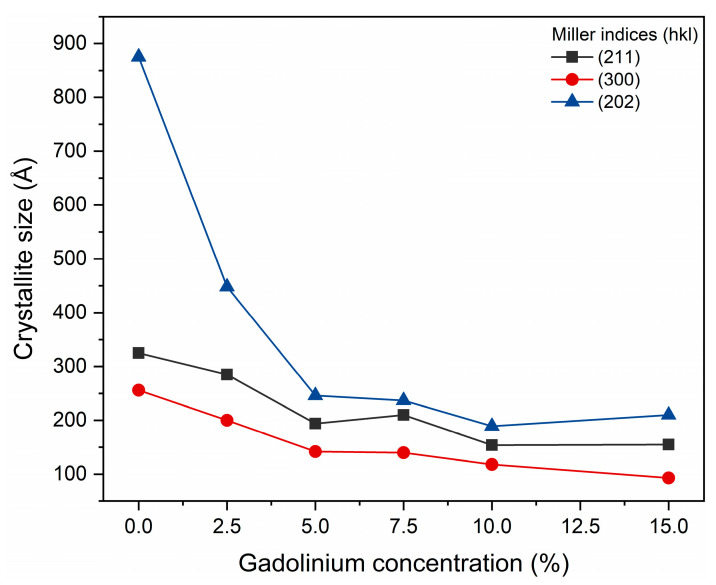
Crystal sizes calculated for planes [202], [211], and [300].

The estimate of the average crystallite size, for the (300), (202) and (211) planes obtained by the JADE/MDI software (Figure 3), demonstrates that as the Gd concentration increases, a reduction in crystallite size occurs. This finding corroborates the structural interpretation extracted from the diffractograms. Previous studies also highlight that interpreting hydroxyapatite diffraction patterns at the nanoscale requires caution. Londoño-Restrepo et al. (2019) [31] demonstrated that biogenic hydroxyapatites exhibit broad, low-intensity XRD peaks not due to low crystallinity, but due to the reduced size of the crystallites. The authors showed that clean bones contain highly ordered nanocrystals and that calcination promotes coalescence and crystal growth, resulting in narrower and more defined peaks. Thus, the broadening of peaks in nanostructured materials may predominantly reflect size effects, and not necessarily lower crystallinity, reinforcing the importance of complementary analyses to distinguish between reduced crystallinity and decreased crystallite size [31].

Taken together, the results of Rietveld refinement and diffractogram analysis indicate that the partial substitution of Ca^2+^ by Gd^3+^ significantly alters the crystal ordering of hydroxyapatite, modifying lattice parameters, while preserving the typical hexagonal structure of the phase. The broad peaks may predominantly reflect size effects, and not necessarily low crystallinity. Such structural changes are consistent with the expectation that dopant ions with different valences and ionic sizes induce local distortions and decrease the average crystallite size.

### 3.2. Transmission Electron Microscopy (TEM)

**Figure 4:**
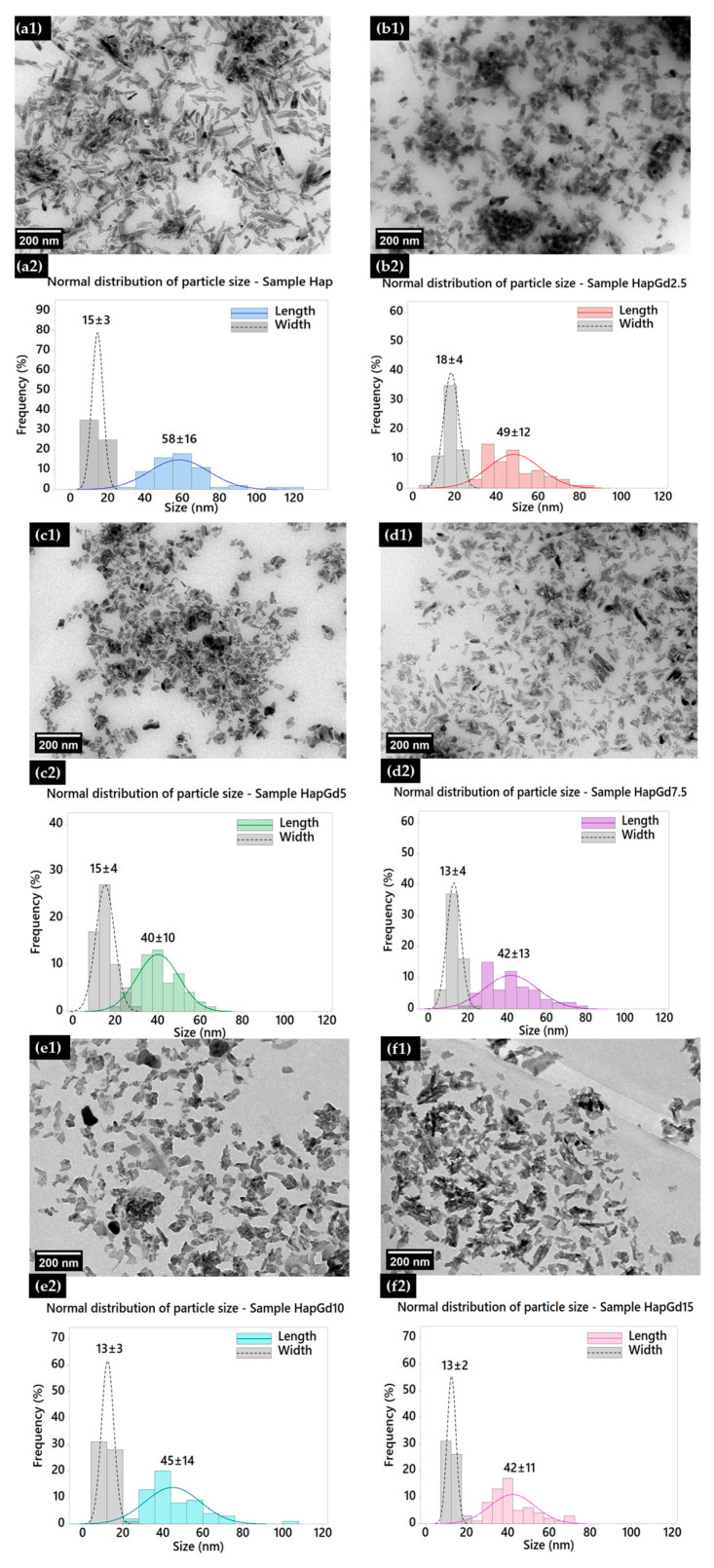
Transmission electron micrographs of (**a1**) Hap, (**b1**) HapGd2.5, (**c1**) HapGd5, (**d1**) HapGd7.5, (**e1**) HapGd10, and (**f1**) HapGd15 samples. Corresponding particle size distributions for (**a2**) Hap, (**b2**) HapGd2.5, (**c2**) HapGd5, (**d2**) HapGd7.5, (**e2**) HapGd10, and (**f2**) HapGd15.

Figure 4 shows TEM micrographs along with the corresponding size distributions (length and width) of pure hydroxyapatite (Hap) nanoparticles and samples doped with different gadolinium contents (2.5 to 15%).

In the Hap sample (Figure 4(a1,a2)), a typical nanorod morphology is observed, which is consistent with materials synthesized through wet processes [32,33,34]. The size distribution shows average values of 58 ± 16 nm in length and 15 ± 3 nm in width, reflecting preferential growth along the c-axis of the crystallographic structure. Upon the initial doping (HapGd2.5, illustrated in Figure 4(b1,b2)), the particles appear to be visibly smaller and less defined, presenting a length of 49 ± 12 nm and a width of 18 ± 4 nm. The decrease in length, coupled with a slight increase in width, suggests that even small amounts of Gd serve as effective perturbations in the anisotropic growth of Hap.

Samples with higher dopant content reinforce this trend. In HapGd5 (Figure 4(c1,c2)), the average length reduces to 40 ± 10 nm, while the width remains relatively constant at 15 ± 4 nm. The marked decrease in the length/width ratio indicates an intensification of structural disorder and an increase in defects related to the incorporation of Gd^3+^. For the intermediate concentration (HapGd7.5, Figure 4(d1,d2)), a greater morphological irregularity is observed, with less defined particles and reduced stability in the crystalline growth process. The average sizes are 42 ± 13 nm in length and 13 ± 4 nm in width, highlighting the onset of a more competitive nucleation regime. In samples with high dopant contents (HapGd10 and HapGd15, Figure 4(e1–f2)), the particles exhibit considerably more isotropic and fragmented characteristics, with granular morphology and almost total loss of the original anisotropy. The length remains reduced (45 ± 14 nm for 10% and 42 ± 11 nm for 15%), while the width stabilizes in the range of 13 ± 2–3 nm. This evolution confirms that the incorporation capacity of Gd^3+^ has reached saturation, with nucleation mechanisms taking precedence over directional growth. The morphological changes observed with increasing Gd^3+^ concentration result from a combination of changes in crystal growth mechanisms and the favoring of nucleation processes. At low to intermediate concentrations, the incorporation of Gd^3+^ preferentially interferes with the anisotropic growth of hydroxyapatite along the c-axis, reducing the length of the nanoparticles, while the width is less affected. At higher Gd concentrations, the presence of the dopant induces a significant increase in structural disorder and defect density, which alters the crystallization kinetics. Under these conditions, nucleation becomes more favorable over oriented growth, leading to the simultaneous formation of multiple crystalline nuclei that compete with one another. This competitive regime interrupts the typical directional growth of hydroxyapatite, resulting in smaller, more isotropic particles with a granular morphology. Therefore, the isotropic morphologies observed at high concentrations of Gd^3+^ do not result from a simple geometric transformation of the particles, but reflect a change in the crystallization regime, in which increased nucleation and suppression of anisotropic growth come to dominate the nanoparticle formation process. These morphological changes reflect a decrease in the average size of the crystallites and an increase in local disorder, factors that contribute significantly to the broadening of the XRD peaks, as shown in Figure 2. Taken together, the results reveal that increasing the amount of gadolinium doping exerts a progressive influence on the morphology and size of the nanoparticles [3,35]. Moderate concentrations lead to a reduction in elongation without complete loss of anisotropy. In contrast, higher levels promote significant disorder, leading to the formation of smaller, more isotropic, and structurally heterogeneous particles. These effects are consistent with Gd^3+^ acting as a disruptive agent for crystal growth, suggesting the existence of an ionic solubility limit in the Hap matrix.

### 3.3. Vibrating Sample Magnetometry (VSM)

**Figure 5:**
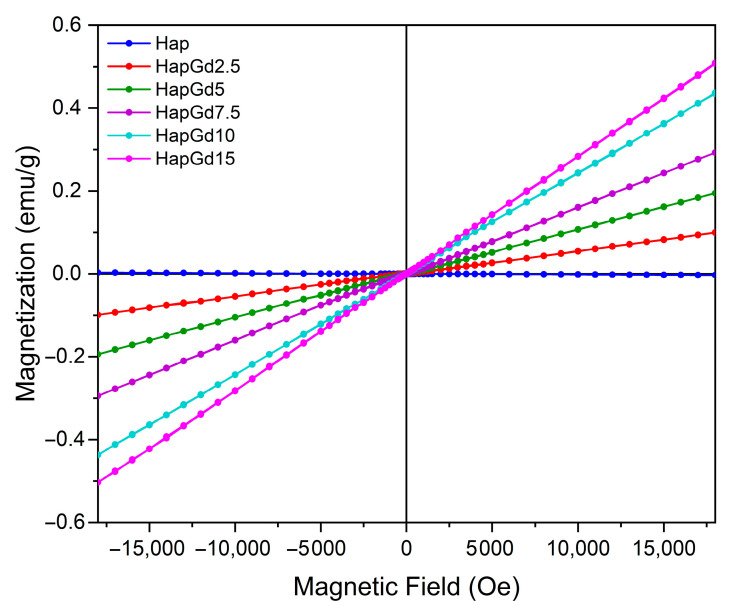
VSM hysteresis loops for Hap, HapGd2.5, HapGd5, HapGd7.5, HapGd10, HapGd15.

Magnetic analysis was performed on pure hydroxyapatite (Hap) samples and compositions doped with various concentrations of gadolinium (Gd^3+^) using vibrating sample magnetometry (VSM). The results revealed a predominantly paramagnetic behavior in all samples containing Gd. Figure 5 shows the hysteresis loops obtained at room temperature. It was observed that as the amount of gadolinium incorporated into the structure increased, the response to the applied magnetic field became more linear.

For undoped hydroxyapatite, a hysteresis curve was observed that is typical of materials with diamagnetic properties and consistent with those widely reported for synthetic apatites [36,37]. The incorporation of Gd^3+^ into the crystal matrix systematically increased magnetization, with the increase proportional to the dopant concentration (see Table 3). It was observed that the coercivity values behaved oppositely to those observed for magnetization. The values decreased with increasing gadolinium content, approaching zero, consistent with the paramagnetic behavior of gadolinium. This evolution of magnetic susceptibility confirms the dominant role of Gd^3+^ ions, whose high magnetic moment arises from their 4f^7^ electronic configuration. For the nanomaterial containing 15% gadolinium, the increase in magnetization was lower than in the other samples. This is attributed to the reduction in crystallite size, which alters the magnetic domain structure and increases structural disorder, with more defects and uncompensated spins [38,39,40]. These changes also result in reduced coercivity, as shown in Table 3. Furthermore, the results suggest that the ions are efficiently distributed in the Hap structure, without the formation of segregated phases rich in Gd that could induce more complex magnetic behaviors.

Similar findings were reported by Xie et al. (2023) [8] for hydroxyapatite nanoparticles co-doped with Si and Gd. In that study, bare hydroxyapatite exhibited characteristic diamagnetic behavior, while samples containing Gd showed a paramagnetic response [8]. Furthermore, the authors observed that magnetization increased proportionally to dopant concentration, highlighting the direct influence of Gd content on the magnetic properties of the material [8]. As in previous studies on the co-doping of Europium and Gadolinium, adding the Gadolinium phase considerably increased the saturation magnetization due to its magnetic properties [41,42].

From an applied perspective, the absence of remanence and coercivity is particularly important, illustrated by sample HapGd15. This ensures that the material does not exhibit residual magnetism after the external field is removed, which is a crucial requirement for safety in clinical applications. Additionally, the ability to modulate the magnetic response by varying the Gd^3+^ concentration enhances the potential of these materials for advanced applications. This includes applications such as contrast for magnetic resonance imaging (MRI), the development of magnetic systems for controlled drug delivery, and the creation of multifunctional platforms for theranostics.

Overall, the results from vibrating sample magnetometry (VSM) demonstrate that Gd^3+^ doping provides an adjustable magnetic response in hydroxyapatite that is compatible with biomedical requirements, without compromising its structural stability or inducing undesirable magnetic behavior.

### 3.4. T_1_ Relaxometry and R_1_ Determination

The longitudinal relaxivities (R_1_) obtained for the nanoparticle formulations and reference samples are summarized in Table 4. The HapGd2.5 nanoparticles exhibited an R_1_ of 2.5 ± 0.1 mM^−1^·s^−1^ at a magnetic field strength of 0.5 T. When functionalized with CMAP-AF, this value nearly doubled to 5.65 ± 0.08 mM^−1^·s^−1^. Notably, the relaxivity of HapGd2.5 CMAP-AF exceeded that of the commercial contrast agent Omniscan™ (4.11 ± 0.1 mM^−1^·s^−1^) under the same experimental conditions. In contrast, the gadolinium-free hydroxyapatite control presented an R_1_ value statistically indistinguishable from zero (−0.01 ± 0.05 mM^−1^·s^−1^), confirming that the observed relaxation enhancement in relaxation is solely attributable to the incorporation of Gd^3+^.

**Figure 6:**
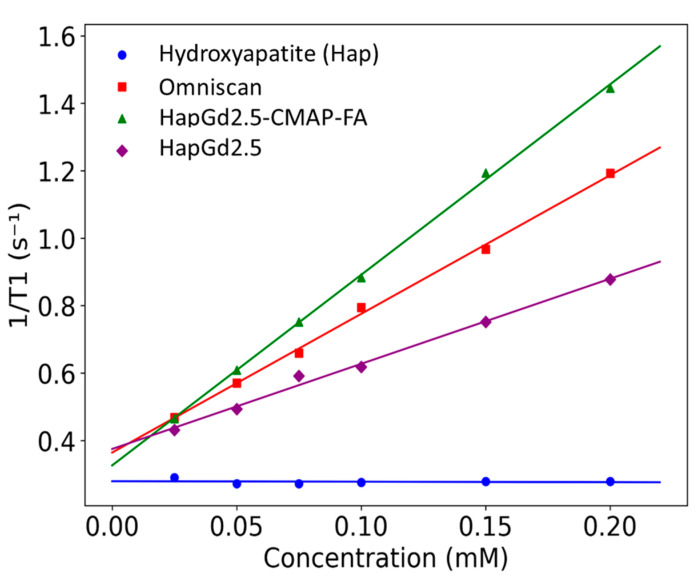
Plot of the longitudinal relaxation rate (R_1_) as a function of Gd^3+^ concentration for Gd-doped nanoparticles and Omniscan™ samples measured at 0.5 T. Symbols represent experimental values, and lines correspond to the linear regression fits.

The substantial increase in R_1_ upon CMAP-AF functionalization suggests that surface modification improves water accessibility to the Gd sites or alters nanoparticle dynamics in a way that promotes T_1_ relaxation enhancement. Although the relaxivities remain within the same order of magnitude as Omniscan™, the CMAP-AF-modified formulation displays performance comparable to, and slightly higher than, the clinical benchmark at 0.5 T (Figure 6). These results indicate that Gd-doped hydroxyapatite nanoparticles—particularly the CMAP-AF–functionalized variant—serve as effective T_1_ contrast-enhancing materials under low-field conditions, while offering a platform adaptable for multimodal or targeted imaging applications beyond conventional small-molecule agents.

Relaxometry analysis was performed only for the sample doped with 2.5% Gd (HapG2.5), since higher gadolinium concentrations imposed significant experimental limitations. At higher concentrations, the nanoparticles exhibited poor colloidal stability and a strong tendency to aggregate. This made it difficult to achieve homogeneous dispersion, even after optimized sonication protocols. Furthermore, the use of high-power sonication, which is necessary for dispersion, could lead to the release of Gd^3+^ ions that were weakly incorporated into the crystal lattice. The simultaneous presence of free Gd^3+^ in solution and Gd^3+^ incorporated into the nanoparticles would generate a heterogeneous system. As a result, the T_1_ value would not accurately reflect the contribution of the doped nanoparticles alone, compromising the reliability of the R_1_ determination. For these reasons, samples with higher doping levels were not included in the relaxometry analysis; however, a detailed study of these samples is planned for the future.

### 3.5. Fourier Transform Infrared Spectroscopy (FTIR)

**Figure 7:**
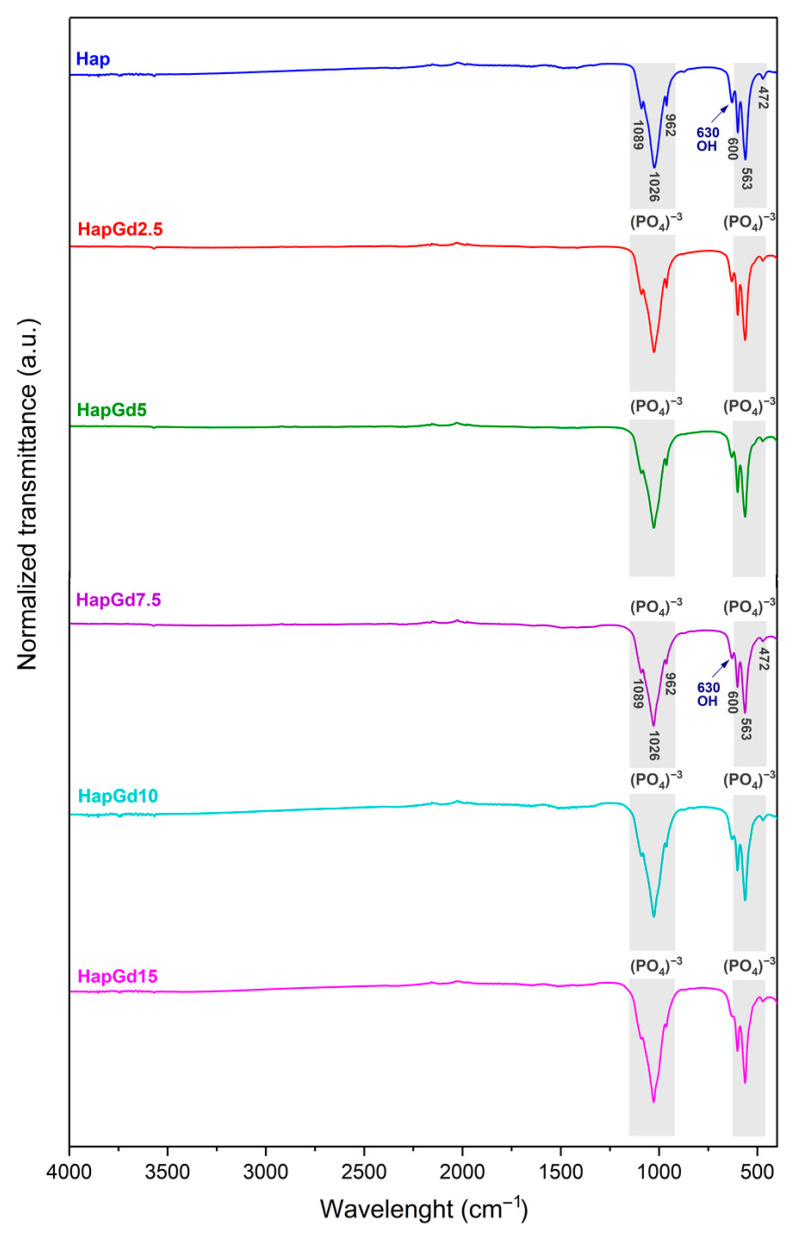
FTIR spectrum of Hap, HapGd2.5, HapGd5, HapGd7.5, HapGd10 and HapGd15 samples.

The FTIR spectra depicted in Figure 7 illustrate the characteristic vibrational bands of both pure hydroxyapatite (Hap) and gadolinium-doped hydroxyapatite samples. This confirms that the intrinsic structural features of the apatite phase are preserved after doping. All samples exhibit a distinct band at 3570 cm^−1^, attributed to the stretching mode of the hydroxyl groups (OH^−^), and another band around 630 cm^−1^, associated with the bending mode of these groups. These bands are consistent with those reported in previous reports on pure and doped hydroxyapatites, as reported by [41,43,44], which indicates that such doping did not eliminate the typical vibrations of the hydroxyl groups.

Additionally, bands found at 472, 563, 600, 962, 1026, and 1089 cm^−1^ correspond to the stretching and deformation vibrational modes of the phosphate group (PO_4_^3−^), which constitute the main spectroscopic signatures of the hydroxyapatite phase. These results match the spectroscopic signatures of crystalline hydroxyapatite and agree with prior studies on rare-earth-doped systems [35,41,44].

Taken together, the FTIR results demonstrate that gadolinium incorporation preserves the main characteristic structural framework of hydroxyapatite. This structural robustness, combined with the optimal performance of the HapGd2.5 sample in magnetic resonance relaxivity measurements, provided a rational basis for selecting HapGd2.5 as the platform for subsequent functionalization steps with curcumin and folic acid. The 2.5% Gd concentration thus represents the optimal balance among effective Gd incorporation, minimal structural disruption, and optimal relaxometric response, making it the most promising core for developing multifunctional nanosystems.

**Figure 8:**
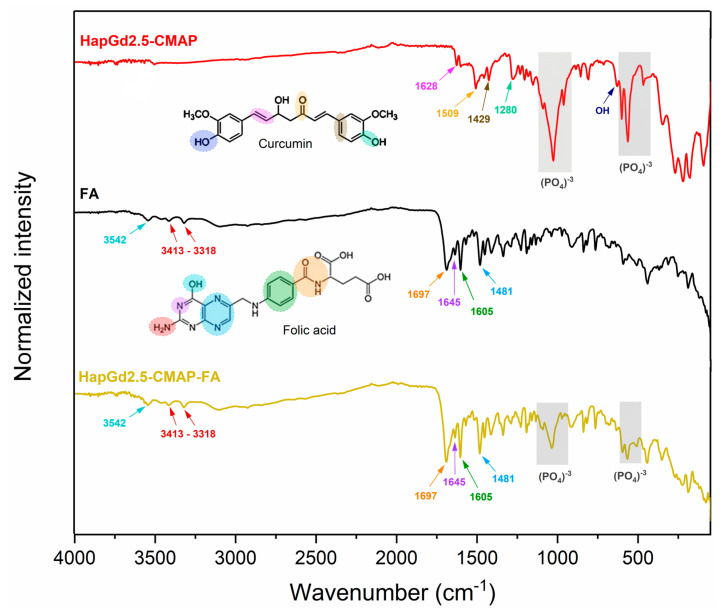
FTIR spectrum of samples HapGd2.5-CMAP, folic acid (FA), and HapGd2.5-CMAP-FA.

Figure 8 presents the FTIR spectra of the HapGd2.5 sample after curcumin addition (HapGd2.5–CMAP) and after subsequent functionalization with folic acid (HapGd2.5–CMAP–FA), alongside the reference spectra of folic acid. The spectrum of the HapGd2.5–CMAP sample exhibited characteristic bands of curcumin, thereby confirming its successful immobilization on the surface of the HapGd nanoparticles. The bands at 1628 cm^−1^, attributed to the C=O vibration conjugated with the aromatic system, and additional bands at 1509, 1429, and 1280 cm^−1^, are related to the aromatic C=C, phenolic C–O, and C–O–C vibrations, respectively, and are particularly noteworthy [45,46,47,48]. The presence of a weak band at 3503 cm^−1^ is consistent with the vibration of phenolic O–H groups of curcumin. Furthermore, the phosphate (PO_4_^3−^) bands, observed around 1040–960 cm^−1^ and 600–560 cm^−1^, remain well defined, indicating that the hydroxyapatite structure was not significantly affected by the functionalization process.

Following the functionalization with folic acid, the spectrum of the HapGd2.5–CMAP–FA sample reveals new bands and enhancements associated with the functional groups of FA, thereby confirming its binding to the surface of the nanoparticle. The bands observed at 3413–3318 cm^−1^ correspond to the N-H vibrations of the pteridine system, while the absorption at 3542 cm^−1^ may be associated with carboxylic and phenolic O-H groups [49]. The characteristic vibrations of FA between 1697 and 1645 cm^−1^ (C=O of carboxylic acid and amide) and the bands at 1605 and 1481 cm^−1^ (aromatic C=C and C=N) are clearly observed in the spectrum of the functionalized nanoparticle, thereby evidencing the presence of folic acid [36]. It is important to note that after the functionalization process, the bands of the group phosphate of the hydroxyapatite remained intact, thereby substantiating the stability of the crystalline structure of the inorganic matrix. The bands previously attributed to curcumina could not be observed, a phenomenon that may be attributable to the superposition of the bands of folic acid.

The findings of this investigation demonstrate that the addition of curcumin and subsequent functionalization with folic acid do not compromise the fundamental structural groups of hydroxyapatite doped with Gd. The observed spectral modifications are solely associated with the functional groups of the organic compounds, thereby validating the effectiveness of the surface modification and preservation of the hydroxyapatite matrix.

### 3.6. Diffuse Reflectance Infrared Fourier Transform Spectroscopy (DRIFTS)

The characterization of functional groups was carried out using DRIFTS. This technique was chosen for its superior precision in investigating the molecular composition and properties of various solid materials, including nanoparticles and heterogeneous systems, since DRIFTS probes the sample as a whole, whereas ATR primarily analyzes only the surface layer. Unlike transmission methods employing KBr pellets, which are prone to variability in preparation, moisture absorption, and sample alterations caused by pressure, DRIFTS requires only the mixing of the sample with a non-absorbing material [50].

For quantitative analysis, the reflectance spectra were converted to Kubelka–Munk (KM) units, which have been shown to exhibit a linear dependence on functional group concentration. This conversion provides a more robust and reliable correlation between band intensity and the surface chemical composition [50].

According to the Kubelka–Munk theory for an infinitely thick layer, the KM function is defined as follows:

f(R∞) = (1 − R∞)^2^/2R∞ = KM, (3)

where R∞ is the absolute reflectance of the infinitely thick sample. The spectrum in Kubelka–Munk (KM) units is expressed as:

KM = k/s,(4)

where ‘s’ denotes the scattering factor and ‘k’ signifies the absorption coefficient, which is defined as k = 2.303ac. Substituting this expression (4) in the equation results in the determination of KM equal to:

KM = 2.303ac/s,(5)

where ‘a’ denotes the absorptivity related to the analyte species and ‘c’ signifies the analyte concentration. The intensity values of KM are directly proportional to concentration when the scattering factor is controlled. This factor provides greater reliability to quantitative analyses [50].

**Figure 9:**
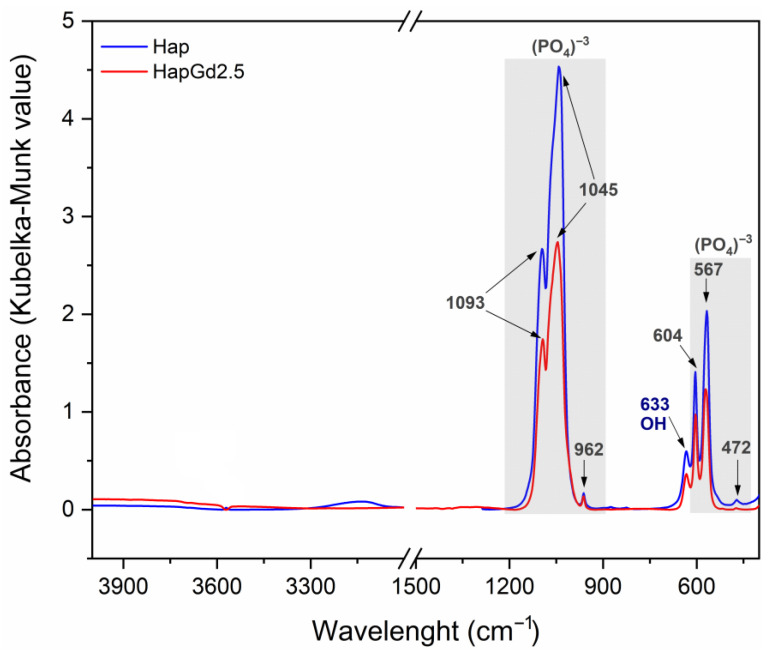
DRIFTS spectra of the Hap (in blue) and HapGd2.5 (in red) samples.

The DRIFTS spectra for samples of pure hydroxyapatite (Hap) and hydroxyapatite doped with 2.5% gadolinium (HapGd2.5) are shown in Figure 9. Both samples exhibit characteristic bands associated with hydroxyapatite, specifically related to the vibrational modes of the phosphate groups (PO_4_^3−^) at 1100–900 cm^−1^ and 600–560 cm^−1^, as well as the vibration of the hydroxyl group (OH^−^) around 630 cm^−1^. The similarity in the spectral profiles suggests that the crystalline structure of hydroxyapatite was preserved after doping, with no clear evidence of secondary phase formation in this spectral region [51]. However, a reduction in band intensity and a slight broadening are observed in the HapG2.5 sample relative to Hap, suggesting increased structural disorder and a possible decrease in crystallinity. This change can be attributed to the partial substitution of Ca^2+^ ions by Gd^3+^, which may introduce vacancies and local distortions in the crystal lattice for charge compensation [52]. This behavior is consistent with the charge compensation mechanism widely discussed in the literature for trivalent cations in apatites. According to research by dos Apostolos et al. (2020), the incorporation of rare earth ions in systems containing Hap has been shown to promote the creation of structural defects, particularly anionic vacancies and local charge redistribution [29]. This phenomenon is crucial to accommodate the valence difference between Ca^2+^ and trivalent dopants.

**Figure 10:**
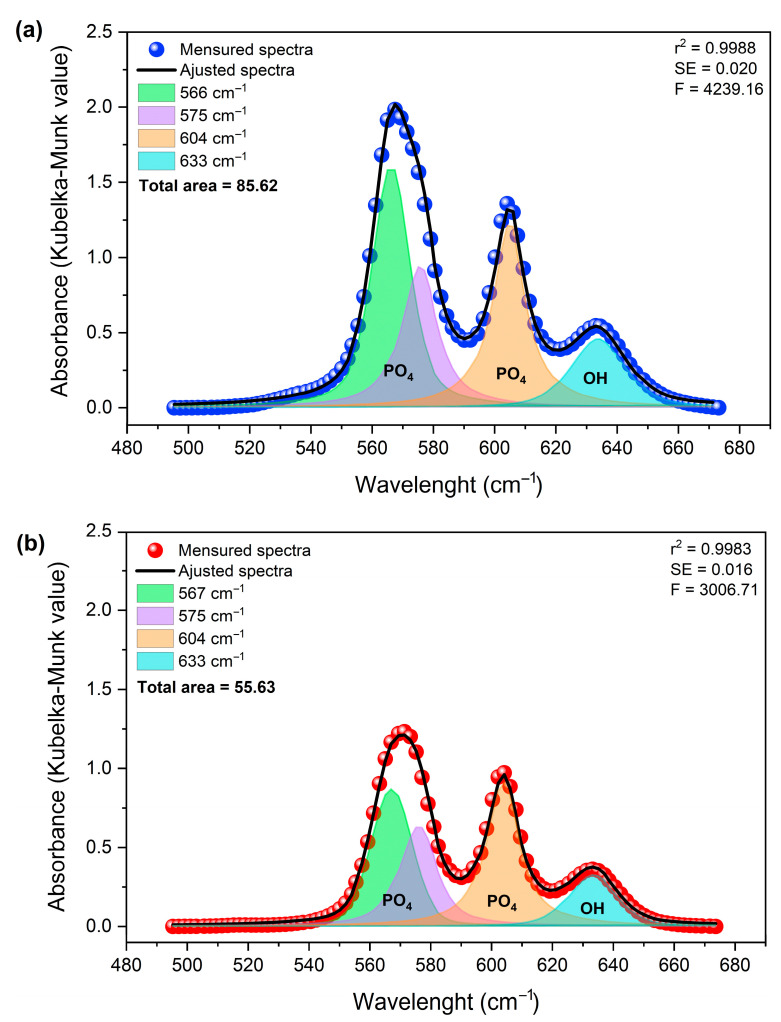
Deconvolution of the DRIFTS spectra of samples (**a**) Hap (in blue) and (**b**) HapGd2.5 (in red) samples in the region of 495 to 675 cm^−1^.

Deconvolution of the spectra in the 495–675 cm^−1^ region (Figure 10) revealed four principal components at approximately 566, 575, 604, and 633 cm^−1^. These components are attributed to the ν_4_ (PO_4_^3−^) vibrational modes and the OH^−^ group contribution, respectively [53].

The approximately 35% reduction in the total integrated area, associated with structurally ordered groups, can be attributed to local perturbations. This finding aligns with observations reported by Mozgova et al. (2025) [54], which highlight the high sensitivity of DRIFTS to changes in surface chemical composition, local symmetry, and subtle structural perturbations in heterogeneous materials. Compared to conventional FTIR, DRIFTS provides improved resolution of variations related to defects, vacancies, and structural rearrangement in particulate solids [54].

The changes observed in the integrated area of the bands, determined by the Kubelka–Munk function (KM = k/s), do not indicate a change in mass. Instead, they suggest changes in the effective concentration of vibrationally active groups in ordered crystalline environments. This finding reinforces the notion that the incorporation of Gd^3+^ exerts a significant influence on the structural coherence of phosphate and hydroxyl sites, without necessarily inducing changes in their primary vibrational positions [55].

For undoped hydroxyapatite, the bands at approximately 567, 575, 604, and 633 cm^−1^ are well-defined and narrow, consistent with a structurally ordered phosphate environment. After doping with 2.5 mol% of Gd, a marked decrease in the total integrated area was observed. This behavior suggests an increase in local disorder, which is probably associated with charge compensation mechanisms necessary for the substitution of Ca^2+^ by Gd^3+^.

In general, the results indicate that doping with Gd^3+^ occurs through substitution in the hydroxyapatite network. This process maintains the fundamental structure of the network while promoting greater local disorder. This behavior is characteristic of cationic substitutions in apatite-based materials and correlates well with the changes observed in the morphology and crystallinity of the particles.

### 3.7. Thermogravimetric Analysis (TGA)

**Figure 11:**
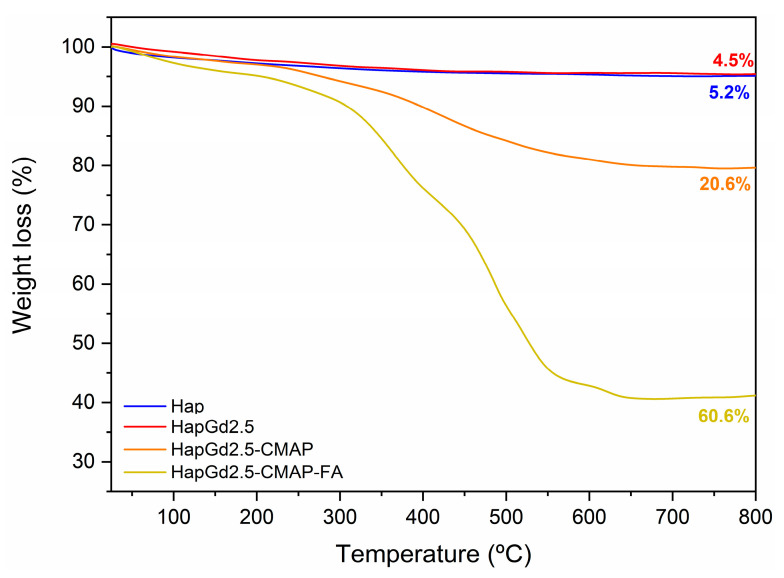
Thermogravimetric curve of the Hap, HapGd2.5, HapGd2.5-CMAP, and HapGd2.5-CMAP-FA samples.

The thermal stability and the degree of functionalization with curcumin and folic acid were evaluated by thermogravimetric analysis (TGA). As shown in Figure 11 and Table 5, both the Hap and the doped HapGd2.5 samples exhibited high thermal stability, with total mass losses of just 4.5% and 5.2%, respectively. These values are consistent with the release of physically adsorbed water and minor residuals from the synthesis route [56], confirming the predominantly inorganic nature of both materials.

In contrast, the functionalized sample HapGd2.5-CMAP displayed a markedly different degradation profile. A well-defined mass-loss event of 20.6% between 215 °C and 700 °C is attributed to the thermal decomposition of curcumin [57,58,59]. This higher loss, when compared to Hap and HapGd2.5, confirms the successful curcumin functionalization and allows for estimating its relative content in the hybrid system.

The co-functionalized sample HapGd2.5-CMAP-FA exhibited an even more pronounced mass loss, reaching 59.4% over the same temperature range. This substantial increase reflects the combined decomposition of curcumin and folic acid, indicating that a large fraction of the material corresponds to organic components effectively anchored onto the nanoparticle surface. The high mass-loss percentage corresponds with the intense vibrational features assigned to both molecules in the FTIR spectra, further supporting the dual functionalization.

Altogether, the progressive increase in mass loss from Hap to HapGd2.5-CMAP-FA directly reflects the sequential addition of curcumin and folic acid. This trend not only evidences the successful functionalization steps but also highlights the multifunctional character of the final material, in which the inorganic hydroxyapatite core is combined with a significant and quantifiable organic fraction tailored for biomedical applications.

It is meaningful to highlight the importance of stability in physiological media, as the decomposition observed by TGA occurs at temperatures much higher than physiological conditions, so the high mass loss does not imply thermal or structural instability in biological environments. Additionally, the presence of organic layers on the surface of the nanoparticles can even act as a stabilizing factor in aqueous media, depending on surface interactions and medium conditions, avoiding the aggregation potential.

## 4. Conclusions

The results demonstrate that microwave-assisted synthesis is an efficient approach for producing Gd^3+^-doped hydroxyapatite nanoparticles, followed by curcumin and folic acid functionalization. The developed material showed high structural stability and functional versatility. XRD analysis showed that Gd^3+^ doping induces significant modifications in the crystalline structure, reducing crystallite size, while maintaining the characteristic hexagonal symmetry of hydroxyapatite. Electron microscopy images corroborated these findings, showing a decrease in nanoparticle size and morphological alterations attributable to the dopant.

DRIFT spectra confirmed the increase in structural disorder in the doped sample, while FTIR and TGA analyses attested to the successful functionalization of curcumin and folic acid, without compromising the integrity of the inorganic matrix. These results demonstrate the efficiency of the functionalization and its compatibility with apatite-based systems.

Magnetic evaluation using VSM and relaxometry revealed that magnetization increases proportionally to the concentration of Gd^3+^ in the structure, confirming the dopant’s role in modulating magnetic properties. Furthermore, the relaxivity measurements of the functionalized nanoparticles were comparable to those of the commercial contrast agent Omniscan™, highlighting their potential as contrast agents for magnetic resonance imaging.

Overall, the physicochemical, structural, and magnetic characteristics obtained highlight the strong potential of these nanoparticles for theranostic applications, integrating diagnostic imaging functionalities and targeted delivery of therapeutic agents. However, despite the promising results, further studies involving advanced relaxometry, as well as in vitro and in vivo biological assays, are necessary to evaluate the efficacy, biocompatibility, and safety of the nanoparticles under physiological conditions. These steps will be fundamental to validating their future use as robust and multifunctional theranostic platforms.

## Figures and Tables

**Table 1:** List of synthesized samples.

Sample	% of Gd in Relation to Ca (mol)	Possible Chemical Formula
Hap	0	Ca_10_(PO_4_)_6_(OH)_2_
HapGd2.5	2.5	Ca_9.75_Gd_0.25_(PO_4_)_6_(OH)_2_
HapGd5	5	Ca_9.5_Gd_0.5_(PO_4_)_6_(OH)_2_
HapGd7.5	7.5	Ca_9.25_Gd_0.75_(PO_4_)_6_(OH)_2_
HapGd10	10	Ca_9_Gd(PO_4_)_6_(OH)_2_
HapGdd15	15	Ca_8.5_Gd_1.5_(PO_4_)_6_(OH)_2_

**Table 2:** Refined unit cell parameters and cell volume of pure Hap and the HapGd samples.

Sample	Unit Cell Parameters		V (Å^3^)	Reliability Factors
	*a* = *b* (Å)	*c* (Å)		
Hap	9.4264 (10)	6.88066 (22)	529.48 (6)	χ^2^ = 2.15 R_w_ = 9.542
HapGd2.5	9.4270 (21)	6.8765 (4)	529.23 (14)	χ^2^ = 4.74 R_w_ = 9.801
HapGd5	9.4265 (22)	6.8745 (3)	529.02 (15)	χ^2^ = 3.25 R_w_ = 7.926
HapGd7.5	9.424 (4)	6.8766 (8)	528.86 (24)	χ^2^ = 4.16 R_w_ = 8.980
HapGd10	9.423 (6)	6.8752 (15)	528.7 (4)	χ^2^ = 4.71 R_w_ = 9.756

**Table 3:** Saturation magnetization and coercivity (Hc) of Hap, HapGd2.5, HapGd5, HapGd7.5, HapGd10, and HapGd15 samples.

Samples	Magnetization (emu/g)	Hc (Oe)
Hap	-	-
HapGd2.5	0.10	176.4
HapGd5	0.19	111.9
HapGd7.5	0.30	106.5
HapGd10	0.44	20.0
HapGd15	0.51	1.8

**Table 4:** Longitudinal relaxivities (R_1_) obtained from the linear regression of 1/T_1_ versus Gd^3+^ concentration for each sample at 0.5 T. Data are expressed as mean ± standard deviation.

Samples	R_1_ (mM^−1^·s^−1^)
Hap	−0.01 ± 0.05
HapGd2.5	2.5 ± 0.1
HapGd2.5-CMAP-FA	5.65 ± 0.08
Omniscam	4.11 ± 0.1

**Table 5:** Weight loss percentages obtained through thermogravimetry analysis of different samples.

			Weight Loss (%)	
Temperature Range (°C)	Hap	HapGd2.5	HapGd2.5-CMAP	HapGd2.5-CMAP-FA
25–150	2.3	2.1	2.5	4.1
150–400	1.9	2.4	7.9	20.4
400–800	0.7	0.7	10.2	36.1

## Data Availability

The original contributions presented in this study are included in the article. Further inquiries can be directed to the corresponding author.

## References

[1] Kansara M., Teng M.W., Smyth M.J., Thomas D.M. (2014). Translational Biology of Osteosarcoma. Nat. Rev. Cancer.

[2] Zhra M. (2025). Advancements in Osteosarcoma Therapy: Overcoming Chemotherapy Resistance and Exploring Novel Pharmacological Strategies. Pharmaceuticals.

[3] Victor S.P., Gayathri Devi M.G., Paul W., Vijayan V.M., Muthu J., Sharma C.P. (2017). Europium Doped Calcium Deficient Hydroxyapatite as Theranostic Nanoplatforms: Effect of Structure and Aspect Ratio. ACS Biomater. Sci. Eng..

[4] Li C., Ding Z., Han Y. (2023). Mn-Doped Nano-Hydroxyapatites as Theranostic Agents with Tumor PH-Amplified MRI-Signal Capabilities for Guiding Photothermal Therapy. Int. J. Nanomed..

[5] Hoveidaei A.H., Sadat-Shojai M., Mosalamiaghili S., Salarikia S.R., Roghani-shahraki H., Ghaderpanah R., Ersi M.H., Conway J.D. (2024). Nano-Hydroxyapatite Structures for Bone Regenerative Medicine: Cell-Material Interaction. Bone.

[6] Iyad N., Ahmad M.S., Alkhatib S.G., Hjouj M. (2023). Gadolinium Contrast Agents Challenges and Opportunities of a Multidisciplinary Approach: Literature Review. Eur. J. Radiol..

[7] Fretellier N., Salhi M., Schroeder J., Siegmund H., Chevalier T., Bruneval P., Jestin-mayer G., Delaloge F., Factor C., Mayer J. (2015). Distribution Profile of Gadolinium in Gadolinium Chelate-Treated Renally-Impaired Rats: Role of Pharmaceutical Formulation. Eur. J. Pharm. Sci..

[8] Xie S., Guo M., Zeng D., Luo H., Zhong P., Deng Z., Wang Y., Xu Z., Zhang P. (2023). Silicon and Gadolinium Co-Doped Hydroxyapatite/PLGA Scaffolds with Osteoinductive and MRI Dual Functions. Front. Bioeng. Biotechnol..

[9] Paterlini V., Bettinelli M., Rizzi R., El Khouri A., Rossi M., Della Ventura G., Capitelli F. (2020). Characterization and Luminescence of Eu^3+^- and Gd^3+^-Doped Hydroxyapatite Ca_10_(PO_4_)_6_(OH)_2_. Crystals.

[10] Roman B., Retajczyk M., Salacinski L., Pelech R. (2020). Curcumin—Properties, Applications and Modificantion of Structure. Mini. Rev. Org. Chem..

[11] Venkatas J., Daniels A., Singh M. (2022). The Potential of Curcumin-Capped Nanoparticle Synthesis in Cancer Therapy: A Green Synthesis Approach. Nanomaterials.

[12] Marinho J.P.N., Gastelois P.L., Macedo W.A.d.A., Cipreste M.F., Sousa E.M.B. Nanostructured System Based on Hydroxyapatite and Curcumin: A Promising Candidate for Osteosarcoma Therapy. Proceedings of the 5th International Caparica Symposium on Nanoparticles/Nanomaterials and Applications.

[13] Jogiya B., Chudasama K., Thaker V., Joshi M. (2018). Synthesis and Characterization of Novel Bio-Material: Nano Composites of Hydroxyapatite and Curcumin. Int. J. Appl. Ceram. Technol..

[14] Sharifi S., Khosroshahi A.Z., Dizaj S.M., Rezaei Y. (2021). Preparation, Physicochemical Assessment and the Antimicrobial Action of Hydroxyapatite—Gelatin/Curcumin Nanofibrous Composites as a Dental Biomaterial. Biomimetics.

[15] Rodrigues M.A., Marinho J.P.N., Vieira L.A.F., Gomes D.A., de Sousa E.M.B. (2024). Internalized Hydroxyapatite Nanoparticles Conjugated with a Modified Form of Curcumin Functionalized with Folic Acid Promote Bone Tumor Cell Necrosis. ACS Appl. Nano Mater..

[16] Ghalehkhondabi V., Fazlali A., Soleymani M. (2022). Folic Acid-Conjugated PH-Responsive Poly (Methacrylic Acid) Nanospheres for Targeted Delivery of Anticancer Drugs to Breast Cancer Cells. J. Mol. Liq..

[17] Luiz M.T., Dutra J.A.P., de Cássia Ribeiro T., Carvalho G.C., Sábio R.M., Marchetti J.M., Chorilli M. (2022). Folic Acid-Modified Curcumin-Loaded Liposomes for Breast Cancer Therapy. Colloids Surf. A Physicochem. Eng. Asp..

[18] Ramezani Farani M., Azarian M., Hossein H.S.H., Abdolvahabi Z., Abgarmi Z.M., Moradi A., Mousavi S.M., Ashra M., Markvandi P., Saeb R.M. (2022). Folic Acid-Adorned Curcumin-Loaded Iron Oxide Nanoparticles for Cervical Cancer. ACS Appl. Bio Mater..

[19] Ghoreyshi N., Ghahremanloo A., Javid H., Tabrizi M.H., Hashemy S.I. (2023). Effect of Folic Acid-Linked Chitosan-Coated PLGA-Based Curcumin Nanoparticles on the Redox System of Glioblastoma Cancer Cells. Phytochem. Anal..

[20] Şimşek Y.E., Avcı Ş. (2019). Synthesis and Characterization of Hydroxyapatite Produced by Microwave Assisted Precipitation Technique. Acta Phys. Pol. A.

[21] Aziz S., Ana I.D., Yusuf Y., Pranowo H.D. (2023). Synthesis of Biocompatible Silver-Doped Carbonate Hydroxyapatite Nanoparticles Using Microwave-Assisted Precipitation and In Vitro Studies for the Prevention of Peri-Implantitis. J. Funct. Biomater..

[22] Marinho J.P.N., Neme N.P., Matos M.J.d.S., Batista R.J.C., Macedo W.A.d.A., Gastelois P.L., Gomes D.A., Rodrigues M.A., Cipreste M.F., de Sousa E.M.B. (2023). Nanostructured System Based on Hydroxyapatite and Curcumin: A Promising Candidate for Osteosarcoma Therapy. Ceram. Int..

[23] Toby B.H., Von Dreele R.B. (2013). GSAS-II: The Genesis of a Modern Open-Source All Purpose Crystallography Software Package. J. Appl. Crystallogr..

[24] Sudarsanan K., Young R.A. (1969). Significant Precision in Crystal Structural Details. Holly Springs Hydroxyapatite. Acta Crystallogr. Sect. B Struct. Crystallogr. Cryst. Chem..

[25] Li Z., Guo J., Zhang M., Li G., Hao L. (2022). Gadolinium-Coated Mesoporous Silica Nanoparticle for Magnetic Resonance Imaging. Front. Chem..

[26] Young R.A., Elliott J.C. (1966). Atomic-Scale Bases for Several Properties of Apatites. Arch. Oral Biol..

[27] Bystrov V.S., Coutinho J., Bystrova A.V., Dekhtyar Y.D., Pullar R.C., Poronin A., Palcevskis E., Dindune A., Alkan B., Durucan C. (2015). Computational Study of Hydroxyapatite Structures, Properties and Defects. J. Phys. D Appl. Phys..

[28] Shannon R.D. (1976). Revised Effective Ionic Radii and Systematic Studies of Interatomic Distances in Halides and Chalcogenides. Acta Crystallogr. Sect. A.

[29] dos Apostolos R.C.R., Cipreste M.F., de Sousa R.G., de Sousa E.M.B. (2020). Multifunctional Hybrid Nanosystems Based on Mesoporous Silica and Hydroxyapatite Nanoparticles Applied as Potential Nanocarriers for Theranostic Applications. J. Nanoparticle Res..

[30] Get’man E.I., Loboda S.N., Tkachenko T.V., Yablochkova N.V., Chebyshev K.A. (2010). Isomorphous Substitution of Samarium and Gadolinium for Calcium in Hydroxyapatite Structure. Russ. J. Inorg. Chem..

[31] Londoño-Restrepo S.M., Jeronimo-Cruz R., Millán-Malo B.M., Rivera-Muñoz E.M., Rodriguez-García M.E. (2019). Effect of the Nano Crystal Size on the X-Ray Diffraction Patterns of Biogenic Hydroxyapatite from Human, Bovine, and Porcine Bones. Sci. Rep..

[32] Wang P., Li C., Gong H., Jiang X., Wang H., Li K. (2010). Effects of Synthesis Conditions on the Morphology of Hydroxyapatite Nanoparticles Produced by Wet Chemical Process. Powder Technol..

[33] Swain S.K., Sarkar D. (2011). Short Communication A Comparative Study: Hydroxyapatite Spherical Nanopowders and Elongated Nanorods. Ceram. Int..

[34] Atta H., Hamdy A., Reyad K., Salim E.I., El E., Shaer A. (2025). El Influence of Hydroxyapatite Nanoparticle Shape on Carrier Concentration and Its Role in Osteosarcoma Cell Inhibition. J. Nanoparticle Res..

[35] Liu Y., Tang Y., Tian Y., Wu J., Sun J., Teng Z., Wang S., Lu G. (2019). Gadolinium-Doped Hydroxyapatite Nanorods as T1 Contrast Agents and Drug Carriers for Breast Cancer Therapy. ACS Appl. Nano Mater..

[36] Cipreste M.F., Peres A.M., Cotta A.A.C., Aragón F.H., Antunes A.D.M., Leal A.S., Macedo W.A.A., de Sousa E.M.B. (2016). Synthesis and Characterization of 159 Gd-Doped Hydroxyapatite Nanorods for Bioapplications as Theranostic Systems. Mater. Chem. Phys..

[37] Saroj S., Vijayalakshmi U. (2025). Structural, Morphological and Biological Assessment of Magnetic Hydroxyapatite with Superior Hyperthermia Potential for Orthopedic Applications. Sci. Rep..

[38] Fe L. (2021). Synthesis, Structural and Microwave Absorption Properties of Cr-Doped Zinc. Ceram. Int..

[39] Jain R. (2023). Effect of Gadolinium Doping on the Structural, Magnetic, Electrical, Optical, and Elastic Properties of Magnetite Nanoparticles. Mater. Sci. Eng. B.

[40] Jacob M., Prinsloo A.R.E., Sheppard C.J. (2026). Results in Materials Particle Size Effect on Structural and Magnetic Properties of Co_0.75_Ni_0.25_Cr_2_O_4_ Composite Nanoparticles. Results Mater..

[41] dos Apostolos R.C.R., Andrada A.D.S., Oliveira A.F., Neto E.S.F., de Sousa E.M.B. (2023). PH-Sensitive Hybrid System Based on Eu^3+^/Gd^3+^ Co-Doped Hydroxyapatite and Mesoporous Silica Designed for Theranostic Applications. Polymers.

[42] Marinho J.P.N., Serrano M.K.S.R.C., Amaral K.P., Oliveira A.F., Vieira L.A.F. (2025). Hydroxyapatite Nanomaterials Co-Doped with Gd^3+^ and Eu^3+^ for Luminescent Imaging and Targeted Drug Delivery. Acad. Mater. Sci..

[43] Somoza M., Rial R., Liu Z., Llovo I.F., Reis R.L., Mosqueira J., Ruso J.M. (2023). Microfluidic Fabrication of Gadolinium-Doped Hydroxyapatite for Theragnostic Applications. Nanomaterials.

[44] Fernandes Vieira L.A., Nunes Marinho J.P., Rodrigues M.A., Basílio de Souza J.P., Geraldo de Sousa R., Barros de Sousa E.M. (2024). Nanocomposite Based on Hydroxyapatite and Boron Nitride Nanostructures Containing Collagen and Tannic Acid Ameliorates the Mechanical Strengthening and Tumor Therapy. Ceram. Int..

[45] Hanafy N.A.N. (2021). Optimally Designed Theranostic System Based Folic Acids and Chitosan as a Promising Mucoadhesive Delivery System for Encapsulating Curcumin LbL Nano-Template against Invasiveness of Breast Cancer. Int. J. Biol. Macromol..

[46] Ismail E.H., Sabry D.Y., Mahdy H., Khalil M.M.H. (2014). Synthesis and Characterization of Some Ternary Metal Complexes of Curcumin with 1,10-Phenanthroline and Their Anticancer Applications. J. Sci. Res..

[47] Perera K.D.C., Weragoda G.K., Haputhanthri R., Rodrigo S.K. (2021). Study of Concentration Dependent Curcumin Interaction with Serum Biomolecules Using ATR-FTIR Spectroscopy Combined with Principal Component Analysis (PCA) and Partial Least Square Regression (PLS-R). Vib. Spectrosc..

[48] Verma G., Gajipara A., Shelar S.B., Priyadarsini K.I., Hassan P.A. (2021). Development of Water-Dispersible Gelatin Stabilized Hydroxyapatite Nanoformulation for Curcumin Delivery. J. Drug Deliv. Sci. Technol..

[49] Mohammed A.S.Y., Dyab A.K.F., Taha F., Abd El-Mageed A.I.A. (2021). Encapsulation of Folic Acid (Vitamin B9) into Sporopollenin Microcapsules: Physico-Chemical Characterisation, in Vitro Controlled Release and Photoprotection Study. Mater. Sci. Eng. C.

[50] Griffiths P.R., de Haseth J.A. (2007). Fourier Transform Infrared Spectrometry.

[51] Mihailova B., Kolev B., Balarew C., Dyulgerova E., Konstantinov L. (2001). Vibration Spectroscopy Study of Hydrolyzed Precursors for Sintering Calcium Phosphate Bio-Ceramics. J. Mater. Sci..

[52] Fadeeva I.V., Deyneko D.V., Barbaro K., Davydova G.A., Sadovnikova M.A., Murzakhanov F.F., Fomin A.S., Yankova V.G., Antoniac I.V., Barinov S.M. (2022). Influence of Synthesis Conditions on Gadolinium-Substituted Tricalcium Phosphate Ceramics and Its Physicochemical, Biological, and Antibacterial Properties. Nanomaterials.

[53] Greene E.F., Tauch S., Webb E., Amarasiriwardena D. (2004). Application of Diffuse Reflectance Infrared Fourier Transform Spectroscopy (DRIFTS) for the Identification of Potential Diagenesis and Crystallinity Changes in Teeth. Microchem. J..

[54] Mozgova O., Chernyayeva O., Sroka-Bartnicka A., Pieta P., Nowakowski R., S.Pieta I. (2025). Physicochemical Characterization of Biodegradable Polymers for Biomedical Applications: Insights from XPS, DRIFT, and AFM Techniques. J. Polym. Environ..

[55] Santos R.D.S., Rezende M.V.D.S. (2014). Atomistic Simulation of Intrinsic Defects and Trivalent and Tetravalent Ion Doping in Hydroxyapatite. Adv. Condens. Matter Phys..

[56] Bulina N.V., Makarova S.V., Baev S.G., Matvienko A.A., Gerasimov K.B., Logutenko O.A., Bystrov V.S. (2021). A Study of Thermal Stability of Hydroxyapatite. Minerals.

[57] Masih R., Iqbal M.S. (2022). Thermal Degradation Kinetics and Pyrolysis GC—MS Study of Curcumin. Food Chem..

[58] Palanisamy G., Lee J., Lee J. (2023). Curcumin-Loaded Hydroxyapatite Nanoparticles for Enriched Removal of Organic Pollutants and Inhibition of Dual-Species Biofilm Formation. Environ. Technol. Innov..

[59] Chattaraj A., Mishra Y., Aljabali A.A.A., Mishra V. (2025). Development and Evaluation of Folic Acid Conjugated Curcumin-Loaded Functionalized Multiwalled Carbon Nanotubes for Enhanced Efficacy in Ovarian Cancer Treatment. Carbon Trends.

